# Correlation between the Bone Mineral Density and Stress on Femur around a Duetto SI Stem

**DOI:** 10.1155/2014/786185

**Published:** 2014-06-26

**Authors:** Rina Sakai, Takeaki Yamamoto, Katsufumi Uchiyama, Kentaro Uchida, Masaki Nakao, Kiyoshi Mabuchi

**Affiliations:** ^1^Department of Medical Engineering and Technology, School of Allied Health Sciences, Kitasato University, 1-15-1 Kitasato, Minami Ku, Sagamihara, Kanagawa 252-0329, Japan; ^2^Department of Orthopaedic Surgery, School of Medicine, Kitasato University, Sagamihara, Kanagawa, Japan; ^3^Graduate School of Medical Sciences, Kitasato University, Sagamihara, Kanagawa, Japan

## Abstract

In cementless stem fixation, BMD reduction around the stem is of concern because it may cause loosening. This BMD reduction is assumed to be caused by stem implantation-induced alteration of the physiological feedback system, which may cause stress shielding and result in loosening, but the causal relationship has not been clarified. In this study, using a Duetto SI stem, we investigated the correlation between the postoperative BMD around the stem and stress. In patients who underwent their first THA at the orthopedic department of our university, the BMD was measured using DEXA, and FEA was performed with an equivalent time course. Time-course changes in the BMD, von Mises stress, and triaxial stress in Gruen zones 1 through 7 were calculated from those measured at 2 weeks and 5 months after surgery. The BMD and von Mises stress showed a bidirectional correlation when Gruen's classification was plotted on the horizontal axis. An increase in stress loaded on bone was assumed to be a factor increasing the BMD. The Duetto SI stem was fixed on the distal side, suggesting its stable fixation. BMD measurement and FEA were useful for quantification of the bone dynamics around the stem from an early phase.

## 1. Introduction

As a complication of total hip arthroplasty (THA), loosening around the hip stem may occur [1]. After cementless fixation, a decrease in the bone mineral density (BMD) around the stem may induce loosening, presenting a problem. Bone has a physiological feedback mechanism by which stress is detected, and the bone mass is controlled to maintain an equilibrium with the bone strength [2]. Hip prosthesis stem implantation can alter this physiological feedback system, reducing the BMD. As a substitute for a marked decrease in the BMD, stress shielding, a term derived from the engineering field, is used [3, 4]. Stress shielding in the physiological feedback mechanism may reduce stress applied to bone, reducing the BMD. However, their causal relationship has not been clarified.

The Duetto SI stem (Cgdb, Italy) has been used for osteoarthritis of the hip associated with congenital acetabular dysplasia by the Department of Orthopedics in our university. Many hip stems previously sold in Japan did not fit the skeleton of Japanese or the deformed bone with osteoarthritis. Thus, the Duetto SI stem was developed as a stem fitting the skeleton of Japanese by Professor Itoman while maintaining the characteristic: conservation of cancellous bone and bone marrow. Therefore, using the Duetto SI stem, we evaluated the correlation between the postoperative BMD and stress around the stem. The time course of the BMD was evaluated in patients who underwent THA in the orthopedic department of our university, and numerical simulation was concurrently performed using a time course equivalent to the clinical time course. Proximal bone loss around a stem, prepared based on the same fixation principle as that of the Duetto SI stem, has been reported [5]. DEXA has been reported to allow the quantitative evaluation of slight BMD changes around the stem after THA [6–8].

Therefore, using the Duetto SI stem, we evaluated the correlation between the postoperative BMD and stress around the stem. The time course of BMD was evaluated in patients who underwent THA in the orthopedic department of our university, and numerical simulation was concurrently performed using a time course equivalent to the clinical time course. The prevention and improvement of stress shielding, which may reduce the fixation force by evaluating the bone and stress states around the stem are indispensable for improving the postoperative results of THA.

## 2. Materials and Methods

This study was approved by the Ethics Committee, Kitasato University School of Medicine (2010-008). We evaluated 35 patients who underwent THA using a Duetto SI stem in the orthopedic department of our university between May 2007 and October 2008 and could be followed up for 5 months or more. The mean age at the time of the operation was 62 years. The underlying disease was coxarthrosis in 30 patients, rheumatoid arthritis in 1, femoral head necrosis in 3, and fracture in 1. The BMD (g/cm^2^) was measured 2 weeks and 5 months after the operation employing an X-ray BMD measurement system (DISCOVERY, Hologic, Inc., USA) using dual energy X-ray absorptiometry (DEXA). Time-course changes in the BMD in zones 1–7 according to Gruen's Classification were calculated [9].

**Figure 1:**
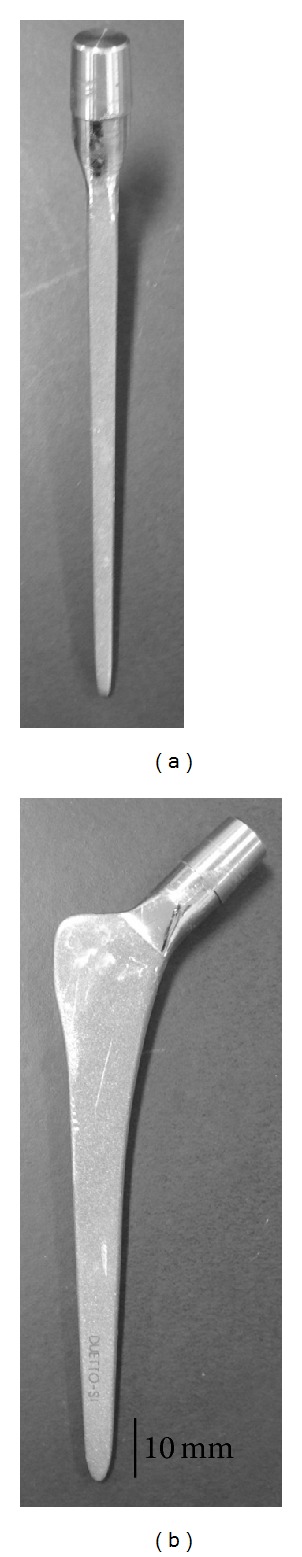
Duetto SI stem with a thin cross-sectional shape. (a) Medial side. (b) Arterial side. The stem surface was processed to produce 6-micron roughness in order to induce bone ongrowth.

The Duetto SI stem (Cgdb, Italy) evaluated in this study was designed to preserve cancellous bone and bone marrow and minimize blood flow impairment in the medullary cavity and has a thin rectangular cross-sectional shape from the neck to the distal end (Figure 1). Since this design is firmly fixed, biting the cortical bone of the femur, it shows superior rotational fixation, which is closely involved in the loosening of cementless stems. The medial curvature of the proximal stem is designed to fit the femoral morphology. The material is titanium alloy (Ti6Al4V), and the stem surface is processed to produce 6-micron roughness in order to induce bone ongrowth.

A model for finite element analysis (FEA) that has 8-node hexahedral isoparametric elements was constructed by altering the shape of the existing hip prosthesis stem [10]. The femoral model was constructed by automatic mesh formation based on CT images of a right femur of a 78-year-old male (OA). The complex system analysis model, which is composed of the stem, cortical bone, and cancellous bone, consisted of 3,087 elements and 4,440 nodes. FE models with about 3,000, 5,000, and 10,000 elements were prepared. Since no marked change was noted in stress values, the model with about 3,000 elements was adopted because the analytical time was shorter. A study using the same model has been reported, in which a mechanical test and analysis were performed to calculate the rotatory displacement of the Duetto SI stem, and the results obtained by the test and analysis were equivalent [11]. Material properties were defined as the combination of the mass density (g/cm^3^), elastic modulus (Pa), and Poisson's ratio [12]. The mass density was determined as 4.5 g/cm^3^ for the stem, 2.3 g/cm^3^ for cortical bone, and 1.9 g/cm^3^ for cancellous bone by substituting values of experimental measurements. Based on the values in the literature, the elastic modulus and Poisson's ratio were set at 110 GPa and 0.29, respectively, for the stem, 15.5 GPa and 0.30 for cortical bone, and 1.00 GPa and 0.33 for cancellous bone [13].

**Figure 2:**
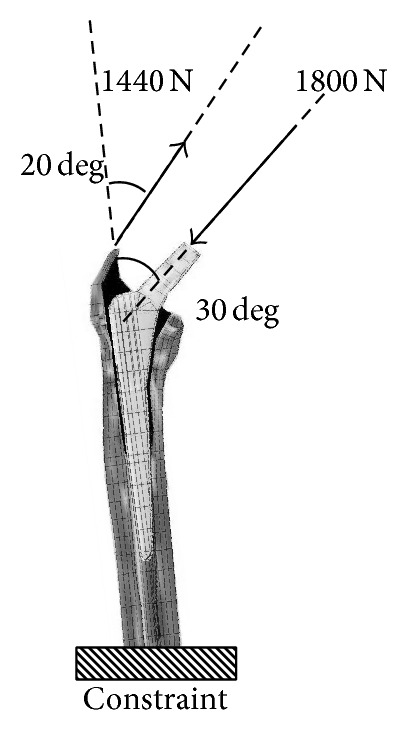
Loading and constraint conditions of FE model. A resultant force of 1,800 N was applied to the head at an angle of 30° along the long axis. An abductor force of 1,440 N was applied from the greater trochanter along the long axis at an angle of 20°. Hatched squares represent restrained regions.

For analysis, Endeavor Pro-4500 (EPSON, Japan) was used as hardware and finite element analysis LS-DYNA Ver.971 (Terrabyte, Japan) as software. The distal end of the femur was constrained in all directions. Based on the ISO7206, a 1,800 N load was applied to the femoral head, and the abduction force was set at 1,440 N [14, 15] (Figure 2). Concerning contact conditions, the coefficient of friction between the cortical bone and the stem was assumed to be 0.3, and automatic control with a consideration of sliding was used [16, 17]. The von Mises stresses and stresses in the 3 axis directions 2 weeks and 5 months after the operation were analyzed, and each value at the middle point of each zone was calculated. The stress value according to the zone in bone around the stem was clarified, and its correlation with the BMD was evaluated.

## 3. Results

**Figure 3:**
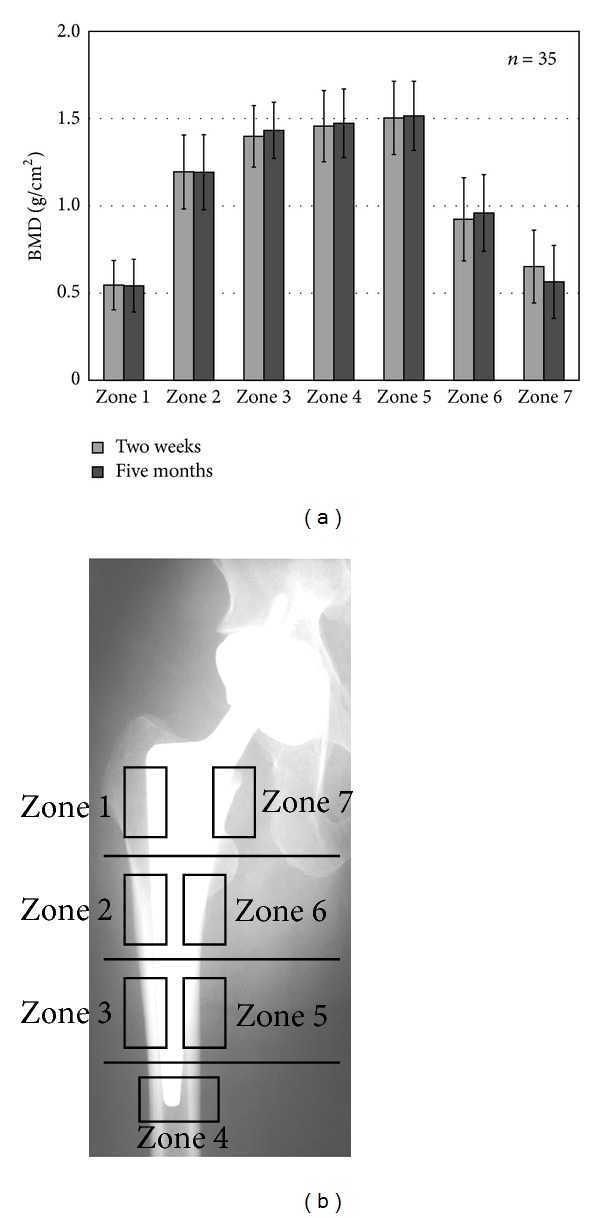
BMD measured by DEXA. (a) BMD values in each zone 2 weeks and 5 months after the operation. (b) X-ray image and Gruen's zone classification.

At both 2 weeks and 5 months after the operation, the BMD was about 0.5 g/cm^2^ in the proximal zones (zones 1 and 7) and about 1.4 g/cm^2^ in the distal zones (zones 3, 4, and 5) of the stem (Figure 3).

**Figure 4:**
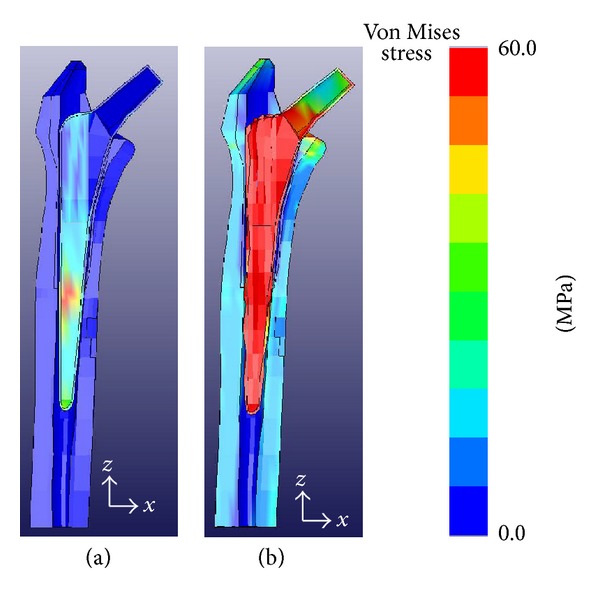
Von Mises stress distribution analyzed by finite element methods. (a) Two weeks. (b) Five months.

**Figure 5:**
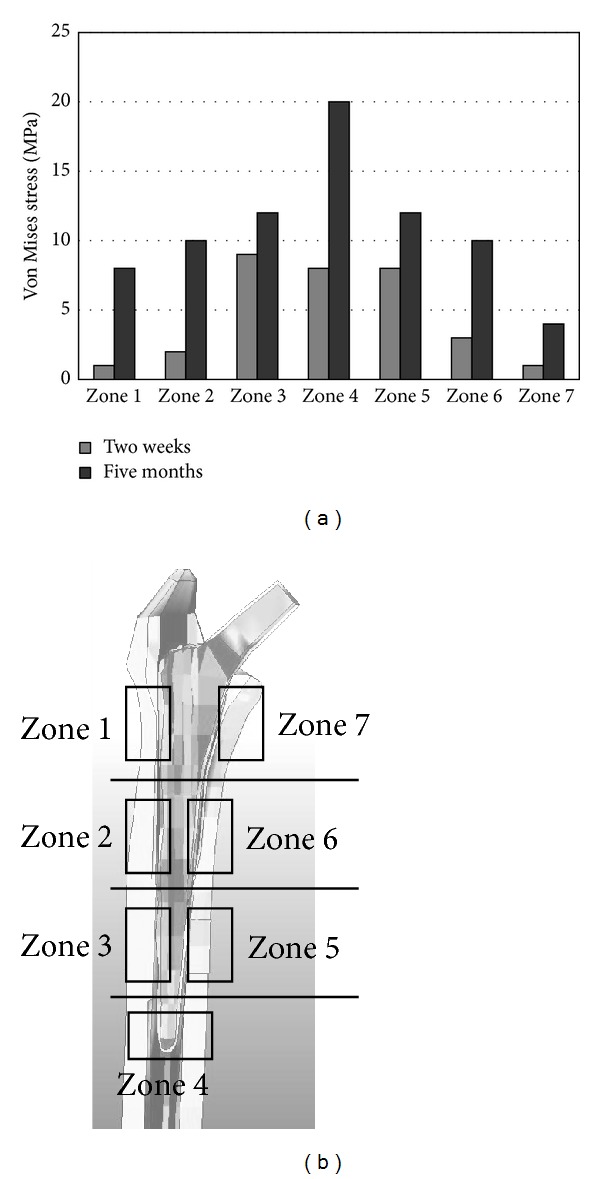
Von Mises stress analyzed by finite element methods. (a) Von Mises stress values in each zone 2 weeks and 5 months after the operation. (b) Finite element model and Gruen's zone classification.

The von Mises stress obtained by FEA 2 weeks after the operation was 9 MPa in zones 3 and 5 and 8 MPa in zone 4 of the femur, and that 5 months after the operation was 20 MPa in zone 4 of the femur (Figure 4). The von Mises stress 5 months after the operation was 4 MPa in zone 7 and ≥8 MPa in the other zones (Figure 5). The von Mises stress 2 weeks after the operation was ≥8 MPa in zones 3, 4, and 5 and that 5 months after the operation was 20 MPa in zone 4. The peak BMD was observed in zone 5, and the peak von Mises stress was observed in zone 3 after 2 weeks and in zone 4 after 5 months. When Gruen's classification was plotted on the horizontal axis, a bidirectional correlation (bell-shaped symmetrical distribution) between the BMD and von Mises stress was observed.

**Figure 6:**
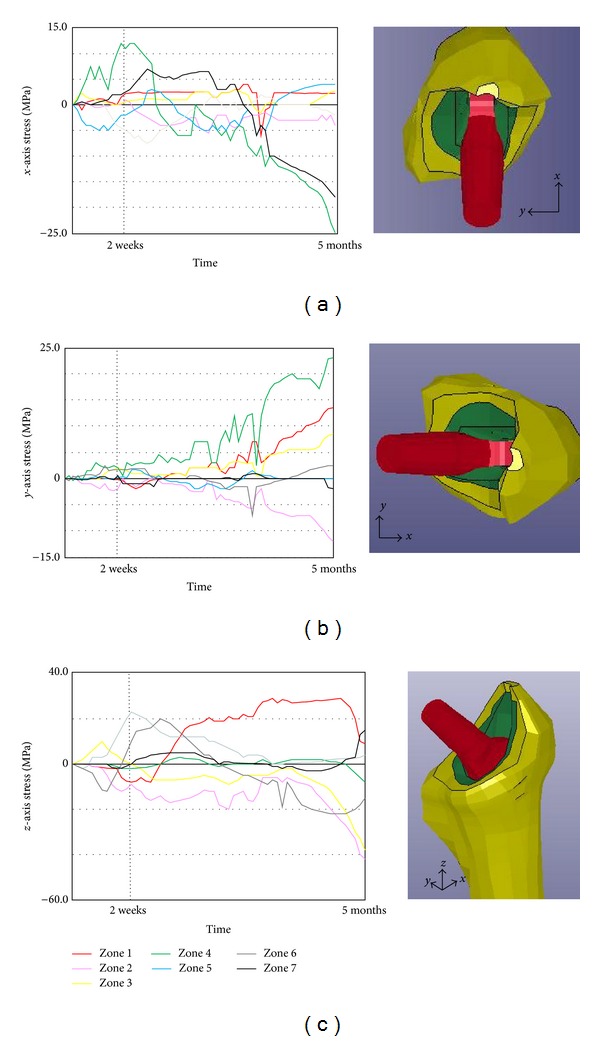
Time series data on stress in the 3 axis directions in each zone obtained by FEM immediately until 5 months after the operation. (a) *x*-axis (medial-lateral) direction. (b) *y*-axis (anterior-posterior) direction. (c) *z*-axis (sinking) direction.

Time series data on stress values in the 3 axis directions in each zone obtained by FEA from immediately to 5 months after the operation showed lower absolute stress values after 5 months than after 2 weeks in some zones (Figure 6). Based on the trend in time series data, the absolute stress value in the *y*-axis direction tended to increase in zones 2 and 4 compared with the other zones. Similarly, in the *z*-axis direction as a sinking direction, the absolute stress value tended to increase in zone 2.

## 4. Discussion

BMD evaluation after cementless THA has frequently been performed [18, 19]. Munting et al. quantitatively measured the BMD after cementless THA in 32 joints of 31 patients during a 6-year postoperative period [20]. Kröger et al. proposed that BMD evaluation should be performed early after the operation [21]. Therefore, we initiated BMD evaluation 2 weeks after the operation. In this study, to clarify bond dynamics around the stem, FEA was performed in combination with BMD measurement by DEXA using an equivalent time course. There have been no studies comparing the BMD obtained by DEXA and the stress obtained by the analysis.

Bone constantly undergoes remodeling, which is the continual cycle of the resorption of hard bone and bone formation to replenish lost areas. Engh and Massin reported that an increase in the BMD is a direct sign of bone formation [22]. Huiskes et al. reported that an increase in stress promotes bone remodeling [23]. When Gruen's classification was plotted on the horizontal axis, the BMD and von Mises stress around the Duetto SI stem showed peaks in the distal area, showing a bidirectional correlation. The results of this study supported the report by Engh and Massin and the proposal by Huiskes et al. Comparison between 2 weeks and 5 months after the operation showed increases in the stress values in the 3 axis directions in many zones with time after the operation. Therefore, an increase in stress on bone may be a factor associated with an increase in the BMD.

The stress value around the Duetto SI stem after 2 weeks was low in the *y*-axis direction. This may have been because of the absence of contact with cortical bone in the anteroposterior direction due to the thin cross-sectional shape as a characteristic of European stems. This gap is useful for preventing blood flow impairment. There was high stress in the *z*-axis direction as the sinking direction, and a distribution indicating a surface force in the axial direction in all zones was observed. The BMD did not differ between 2 weeks and 5 months after the operation, but the stress values in the 3 axis directions were slightly higher after 5 months than after 2 weeks. This may have been because surface force in the axial direction and circumferential stress occurred due to stem sinking into the medullary cavity. There have also been many clinical reports of sinking using other stems [24].

Using another Zweymueller type of stem, stress shielding in the proximal area was reported [25]. Using the Duetto SI stem, since stress based on MPa values was observed in each zone of bone and the entire stem, stress shielding was unlikely. Engh and Massin described that the stem tip is stable when the BMD is high in zone 4 [22]. Thus, the fixation force of the Duetto SI stem may be obtained in the distal area, which suggests stable fixation.

Concerning the BMD after cementless THA, Nakamura observed 25 joints of 21 patients who underwent an operation using an Omniflex stem from 1 month until 1 year after the operation and reported low BMD values in zones 1 and 7 [26]. The results of this study were consistent with theirs. Due to constant metabolism-associated changes in the BMD, the observation period should be increased. In this study, since FEA was concurrently performed using an equivalent time course, the observation period was 5 months due to the limit of machine specifications. In the future, a long-term follow-up of the course of the BMD will be necessary. In addition, to obtain more reliable data, the results of analysis should be evaluated using statistical methods with a consideration of errors. Regarding the limitations of this study, since von Mises stress is affected by the bone modulus, it is necessary to prepare several models by changing the bone modulus. Simplified cortical and cancellous bones were used, but more accurate investigation is possible using a model to which tendons, ligaments, and muscles are added.

Evaluation of the BMD and stress around the Duetto SI stem revealed the following.

1. There was a bidirectional correlation between the BMD and stress when Gruen's classification was plotted on the horizontal axis.
2. The Duetto SI stem was fixed on the distal side, which suggested stable fixation.
3. BMD evaluation and FEA were useful for quantitatively clarifying bone kinetics around the stem.
